# US and CT of 2 pediatric cases of self-inflicted pneumoparotid and cervicofacial emphysema

**DOI:** 10.1016/j.radcr.2025.01.055

**Published:** 2025-01-31

**Authors:** François Chalard, Méryle Laurent, Eugénie Barras, Seema Toso

**Affiliations:** Pediatric Radiology, Hôpitaux Universitaires de Genève, Genève, Switzerland

**Keywords:** Pneumoparotid, Children, Ultrasonography, CT

## Abstract

Pneumoparotid is a rare cause of parotid swelling characterized by the presence of air in the Stensen's duct and/or the parotid gland. In children, it is often self-inflicted, due to psychiatric disorder or recreational habits (puffing the cheeks, blowing balloons, bruxism…). The diagnosis may be suggested by specific signs such as crepitus or foamy saliva flowing from the Stensen's duct. Here, we present 2 cases of involuntary self-inflicted pneumoparotid, diagnosed by ultrasound and CT, in a child and a teenager. Imaging is useful for diagnosis and excluding complications. Ultrasonography may be sufficient to make the diagnosis of pneumoparotid but is less sensitive than CT. CT can make the diagnosis, but also more completely explores the deep cervical spaces and the thorax, especially important in cases with respiratory symptoms. Treatment is usually conservative, in association with behavioral counselling or psychologic/psychiatric therapy, to avoid activities leading to intra oral pressure increase.

## Introduction

Pneumoparotid is a rare cause of parotid swelling, the causes of which are mainly infectious, inflammatory or tumoral in pediatrics. Here, we present 2 cases of involuntary self-inflicted pneumoparotid diagnosed by ultrasonography (US) and/or CT in a child and a teenager. We briefly review the causes, clinical presentation, imaging modality and treatment of this little-known disorder.

## Cases

### Case 1

**Fig. 1:**
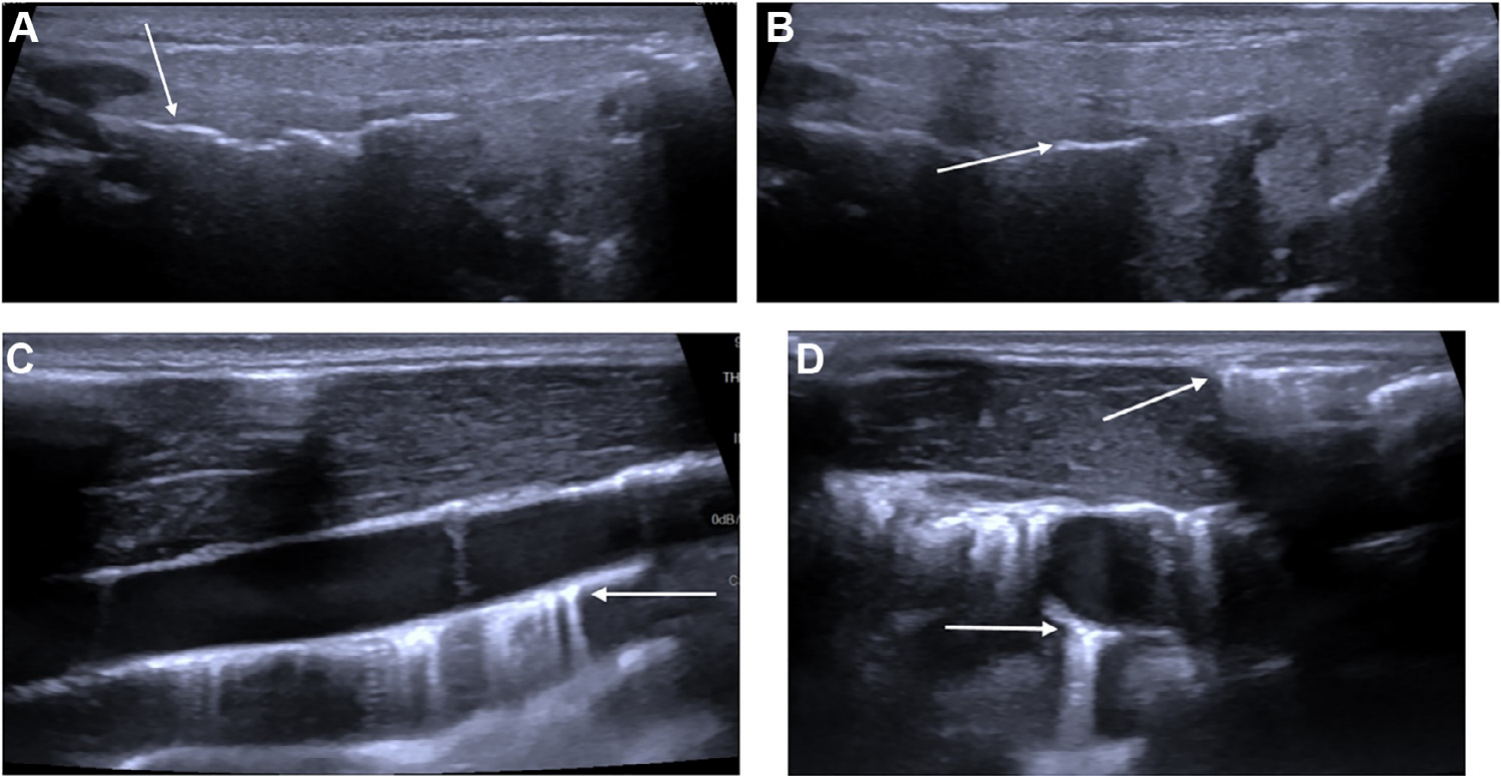
US showing air bubbles in both parotid glands (A and B) and emphysema in subcutaneous fat and deep cervical spaces (C and D).

A 5-year-old girl consulted the emergency room for bilateral, painless cervico-facial swelling, predominantly in the parotid regions. There were no local inflammatory signs. She did not have fever, dysphagia, pharyngeal inflammation or dyspnea. Lung auscultation gave normal results. Her medical history was relevant for left hemiparesis after a perinatal cerebral hemorrhage in the neonatal period linked to an extremely premature birth, which required subdural peritoneal drainage. Cervico-facial US revealed bilateral pneumoparotid and cervical emphysema (Fig. 1). The thyroid gland could not be visualized because of the large amount of subcutaneous emphysema. No air bubbles were seen along the trajectory of the ventriculoperitoneal drain in the neck. Internal jugular veins were permeable. No adenomegaly was seen.

Because of no other obvious finding, the working hypothesis of emphysema and pneumoparotid resulting from increased intraoral pressure was suggested. In the absence of respiratory symptoms and dysfunction, no further imaging studies were performed. In that the clinical tolerance was excellent, no treatment was prescribed and the girl returned home. Complete, spontaneous regression of symptoms occurred quickly.

**Fig. 2:**
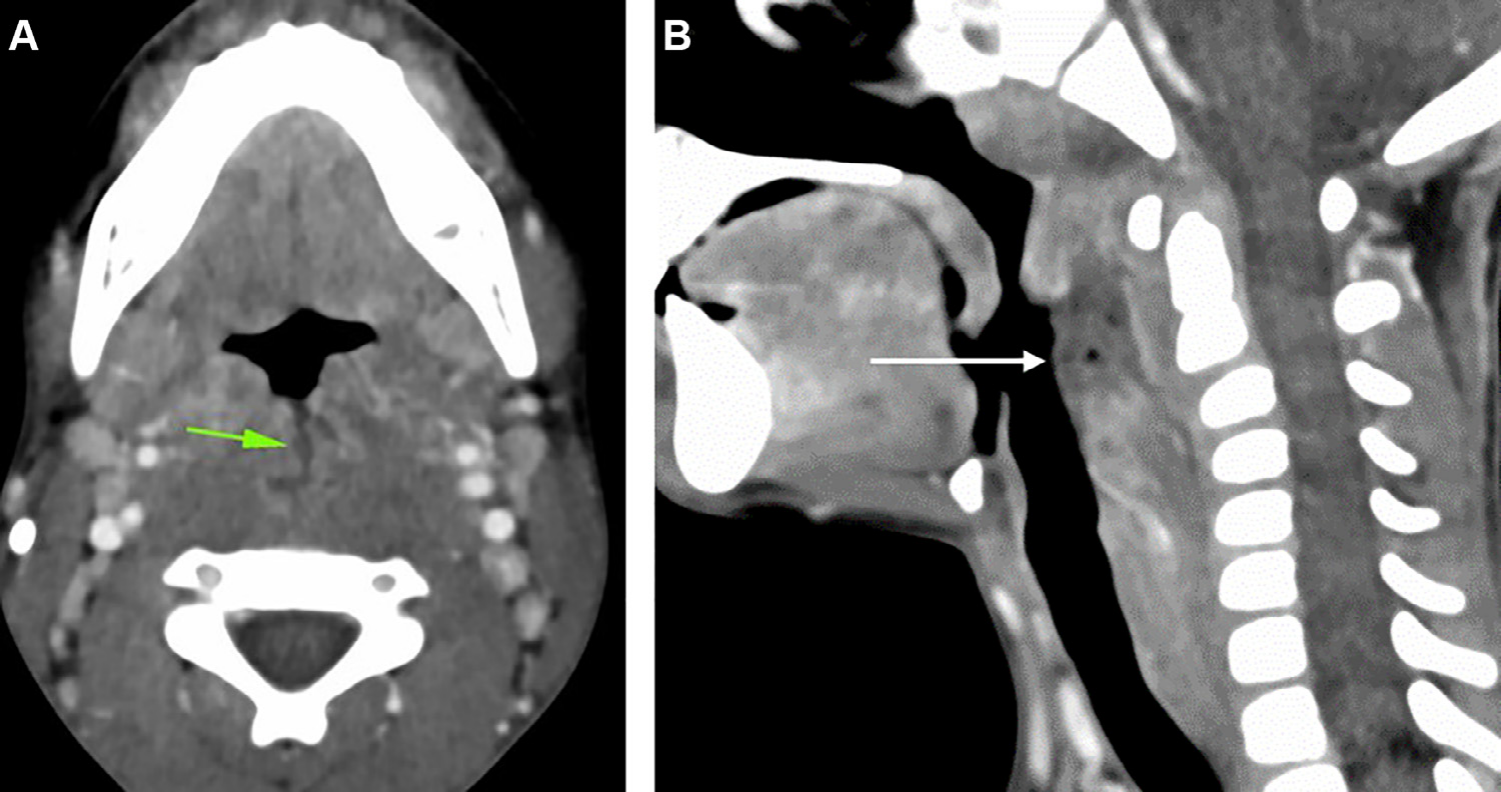
CT showing a median breach of the oropharyngeal mucosa, contiguous with a collection of retropharyngeal fluid containing air bubbles (A and B).

One month later, the girl returned to the emergency room because of odynodysphagia, torticollis and fever. Oral examination revealed scarring of the soft palate, asymmetric swelling of the tonsils and a labial crusted lesion. No respiratory symptoms were observed. Mild biological inflammatory syndrome was evidenced but no leukocytosis. Cervico-facial CT scan was ordered to characterize and evaluate the extension of the lesions. CT scan revealed a large median breach of the oropharyngeal mucosa, continuous with collection of retropharyngeal fluid containing air bubbles (Fig. 2). There was no foreign body in the collection nor peripheral enhancement of this collection. Additionally, no signs of nonaccidental injury were found.

Surgical exploration revealed a large ulceration of the entire oropharynx, with a chronic appearance, and a pedunculated lesion of the left palate tonsil. This pediculated lesion was biopsied and the pathological diagnosis was a fibroepithelial polyp. Immunohistochemistry excluded a tumor, and meticillin-sensitive *Staphylococcus aureus* was found in the retropharyngeal collection. No virus was found in the labial crusty lesion.

The girl was given intravenous antibiotics (co-amoxicillin) and painkillers. Two weeks later, she had no symptoms, and oral examination revealed incomplete regression of the pharyngeal ulceration. An interview with her mother revealed that although the girl did not have known behavioral disorders, she would sometimes play with a straw in her mouth and puff out her cheeks. In addition, the second episode was hypothesized to be post-traumatic, self-inflicted, and complicated by bacterial infection.

### Case 2

A 13-year-old girl consulted a physician outside the hospital for spontaneous swelling of the left parotid gland and very mild fever (temperature 37.8°C). The initial diagnosis was parotitis, and the treatment was oral antibiotics (amoxycillin). The day after, the girl consulted our emergency room because the facial swelling had suddenly extended to the ipsilateral cheek and upper eyelid. On palpation, the swelling was hard and painful, although the skin was unchanged. Oral examination did not reveal any significant anomalies, notably Stensen's duct termination. There were no respiratory symptoms or fever. Ophthalmological examination revealed only eyelid thickening. Laboratory test results were normal (no inflammatory syndrome or leukocytosis). US revealed an enlarged, ill-defined and heterogeneous left parotid gland and hyperechogenicity of the subcutaneous fat but no clear evidence of air bubbles. In addition, there was no dilatation of Stensen's duct, no salivary lithiasis and no fluid collection. No clear diagnosis was proposed, parotitis or preseptal cellulitis being unlikely at that time. Antibiotic treatment was continued.

**Fig. 3:**
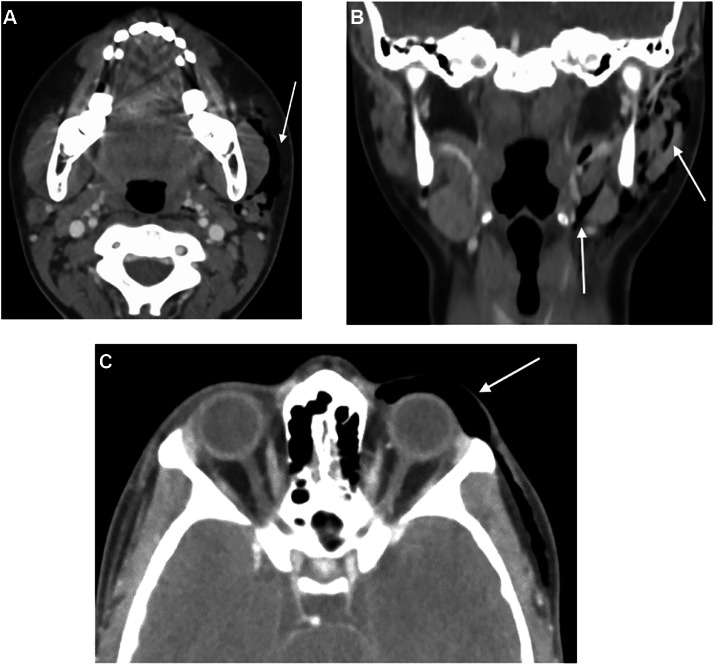
Aaxial and coronal CT showing left pneumoparotid, palpebral and cervical emphysema.

The day after, 2 days after the onset of the symptoms, CT scan revealed left pneumoparotid, pneumorbit and cervico-facial emphysema in the subcutaneous fat of the eyelid and in deep cervical spaces (Fig. 3). No other anomalies were detected, in the salivary glands, pharynx, lymph nodes, muscles or bones. Medical history did not reveal trauma or wind instrument use but rather depression, obsessive-compulsive disorder and bruxism. So, after excluding other differential diagnoses, we concluded that the symptoms were self-inflicted, by bruxism or another abnormal maneuver (self-inflicted biting, sucking movements) leading to increased intraoral pressure and air flow from the outside to Stensen's duct, face and neck.

**Fig. 4:**
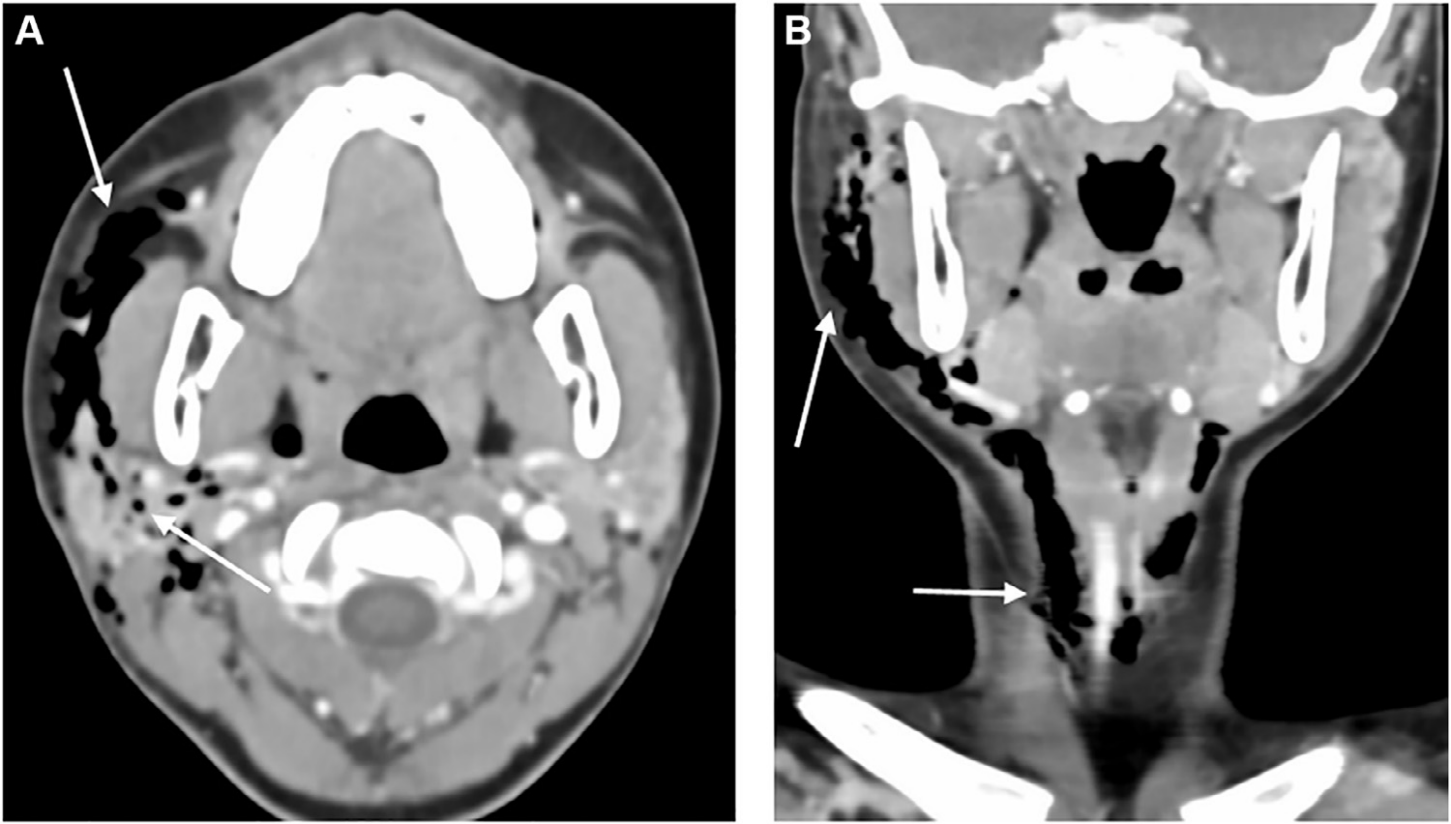
A axial and coronal CT showing recurrence of the pneumoparotid and cervical emphysema, on the right side.

The symptoms regressed completely in 2 or 3 days, but 3 weeks later, the patient consulted the emergency room for contralateral recurrence of the cervico-facial swelling. CT scan revealed right pneumoparotid (superficial and deep lobes involved) and cervico-facial emphysema and an almost complete regression of the former emphysema and contralateral anomalies (Fig. 4). Laboratory findings were normal. The treatment was antibiotics and a nonsteroidal antiinflammatory drug, and symptoms regressed within a couple of days.

## Discussion

Pneumoparotid, first described by Hyrtl in 1865, is defined by the presence of air in the parotid gland and/or Stensen's duct [1]. It results from retrograde insufflation of air through Stensen's duct. This reflux of air is the consequence of both an anatomical anomaly, incompetence of the antireflux system (patulous ostium of Stensen's duct, hypotonia of the buccinator muscle, hypertrophy of the masseter muscle) and elevated intraoral pressure [2,3]. The disorder is most commonly observed in teenagers and young adults. In the general population, the leading causes of excessive intraoral pressure leading to pneumoparotid are self-infliction, somatic diseases and iatrogenic procedures, the etiology of the pneumoparotid remaining unidentified in 20%-24% of patients [[4], [5], [6]].

The self-infliction can be intentional or not. Nonintentional self-inflicted pneumoparotid is due to abnormal habits, including glass blowing (historical occupational disease in the 20th century), playing a wind instrument [7], blowing balloons [8], diving or blowing bubbles underwater [9], preventing oral irritation due to an orthodontic brace [10] or stopping aphthous ulcer pain [11]. Intentional self-induced pneumoparotid is often linked to psychiatric or behavioral disorders [12,3]. Thus, self-inflicted pneumoparotid has been reported in children or teenagers who tried to avoid going to school [13], as some French foreign legionnaires did, blowing hard in a bottle, to avoid duty during World War I [14].

The main somatic diseases leading to pneumoparotid are obstructive sleep apnea syndrome [15], coughing attack [16], head and maxillofacial trauma [17].

Iatrogenic causes are various and their incidence has increased since the 1990s [4] spirometry [18], positive pressure ventilation in the intensive care unit [19], long-term use of oronasal continuous positive airway pressure [20], dental procedures [21], upper endoscopy [22], and a mandibular advancement device for obstructive sleep apnea [23].

The clinical signs can be unilateral or bilateral, in one third of cases [4,24]. They can be repetitive over the years until the diagnosis is established [25]. The most common symptom is acute swelling of the parotid gland, which may be painless, uncomfortable or mildly painful [5]. In the absence of complications, the overlying skin is normal, there is no fever and no lymphadenomegaly, and the general condition is well preserved. Other clinical signs, less common, must be looked for: crepitus or a sensation of crackling in the parotid region, foamy saliva flowing from the orifice of Stensen's duct when pressing on the parotid gland, oral sounds [15] and emphysema in the face, neck or mediastinum, which is less frequent and inaccessible to clinical examination in the latter location [4,25]. When the air is collected in a large bubble within the parotid gland [26], there is no crepitus and the swelling is firm or even hard, mimicking a true parotid mass [27,3].

Biologically, there may be hyperamylasemia and a mild inflammatory syndrome, secondary to pneumatic dissection of the gland with release of its amylase [28].

In children, the most common differential diagnosis of parotid gland swelling is an inflammatory/infectious process, followed by tumors, first branchial cleft cysts and systemic disease [29]. Infection by gas-forming organisms may be difficult to differentiate, but the marked discrepancy between the degree of parotid swelling, relative general well-being and limited clinical findings suggests the diagnosis [28].

Regarding differential diagnoses of pneumoparotid and cervical emphysema, the causes of gas bubbles in the cervical spaces can be differentiated depending on whether the gas is produced in situ, is from air-containing structures (tracheobronchial tree, sinuses or esophagus), or is introduced from the outside. Gas bubbles may be produced in situ in cases of necrotizing fascitiis, a rare bacterial infection (*S. aureus*, group A Streptococcus) of the subcutaneous tissues, fascia and surrounding soft tissue structures. This disease with significant local signs (edema, erythema, crepitus, necrosis) is potentially fatal and rapidly progressing, which clinically differs greatly from pneumoparotid with cervical emphysema. Biologically, there is an inflammatory syndrome, hyperleukocytosis. In addition to gas bubbles, imaging may reveal fascial thickening, cloudy fluid collection or abscess, and swelling of subcutaneous soft tissue [30].

Gas bubbles can come from air-containing structures, most commonly the tracheobronchial tree. During asthma, there is increased pressure in the upper respiratory tract, which can cause alveolar rupture. Then, air flows from the alveolus to the pulmonary interstitium and sometimes leads to a pneumomediastinum and cervical emphysema [31].

Exceptionally, air bubbles can be introduced from the outside, as in case of compressed air blast injury [32]. Therefore, accidental circumstances are the key to diagnosis.

### Imaging modalities for diagnosis

US is a useful tool to detect pneumoparotid [[33], [34], [35]] and superficial cervical emphysema. Indeed, there is a discontinuity of the parotid capsule (the superficial layer of the deep cervical fascia) in its superomedial part, allowing potential passage of air to the parapharyngeal and retrophryngeal spaces [36]. The air can also flow from the parotid gland to cervical deep spaces via acinar and capsular rupture. Pneumoparotid and cervical emphysema appear as hyperechoic lines or spots, also known as a “comet-tail” artefact. In our first case, the artefact was so great that the thyroid gland was hidden. However, a small amount of air can be missed by this technique, especially in deep cervical spaces, as in our second case.

CT is the gold standard imaging to diagnose pneumoparotid, being more sensitive than US, because very small bubbles of gas are easily detected. CT also allows for an overview of all cervical spaces, even deep ones [28,37,38]. Cervical and thoracic CT is mostly useful and recommended in case of respiratory symptoms, to look for extensive emphysema [39] and potentially life-threatening conditions: bronchial, pulmonary or esophageal disorder and pneumothorax or pneumomediastinum [40,41]. Indeed, pneumoparotid is sometimes the tip of the iceberg and accompanies life-threatening conditions.

Cases of pneumoparotid diagnosed by sialography have been reported [33], but this technique is limited to the exploration of Stensen's duct and the parotid canaliculi and does not show the parotid parenchyma.

MRI may occasionally show pneumoparotid, but it is rarely available in an emergency, cannot distinguish calcification from small bubbles of gas well and does not allow for an appropriate exploration of the thorax (when necessary) [42].

Recurrent parotid insufflation is not entirely benign and may predispose to sialectasias, recurrent parotitis, and extensive subcutaneous emphysema, with possible airway compression [43]. Pneumoparotid associated with parotitis is called pneumoparotitis [36,4].

The outcome is most often favorable, and conservative treatment generally yields good results. Treatment should be targeted to the cause of the pneumoparotid. In children, because pneumoparotid is often self-inflicted, psychiatric or psychologic therapy and patient education are of the utmost importance, to avoid recurrence [44]. Antibiotics, anti-inflammatory drugs, parotid gland massage, warm compresses or sialagogues may be used in acute cases, to treat or prevent complications such as parotitis [3]. Surgery is rarely needed and is reserved for severe cases of chronic or recurrent disease, with Stensen's duct ligation, ductoplasty or parotidectomy [2,5].

## Conclusion

Pneumoparotid is a rare cause of parotid swelling characterized by the presence of air in Stensen's duct and/or the parotid gland, on one or both sides. In children, it is often self-inflicted, due to a psychiatric disorder or recreational habits (puffing the cheeks, blowing balloons, bruxism etc.). Such behavior may not be discovered initially and should be investigated carefully, with repeated questioning of the child and parents. The diagnosis may be suggested by specific signs such as crepitus or foamy saliva flowing from Stensen's duct. Imaging is useful for diagnosis and excluding complications. US may be sufficient for the diagnosis but is less sensitive than CT. CT can be used to establish the diagnosis but also more completely explores the deep cervical spaces and the thorax, especially important in cases with respiratory symptoms. Treatment is usually conservative, in association with behavioral counselling or psychologic/psychiatric therapy, to avoid activities leading to increased intraoral pressure.

## Patient consent

We, the authors, certify that we have obtained (for the family of each patient) written and informed consent for publication of their case.
